# Wind farm noise negatively impacts the calling behavior of three frogs in Caatinga dry forests

**DOI:** 10.1371/journal.pone.0318517

**Published:** 2025-03-19

**Authors:** Rogério Ferreira de Oliveira, André Felipe de Araujo Lira, Valentina Zaffaroni-Caorsi, Matheus Leonydas Borba Feitosa, Geraldo Jorge Barbosa de Moura

**Affiliations:** 1 Programa de Pós-graduação em Ecologia, Departamento de Biologia, Universidade Federal Rural de Pernambuco, Recife, Brazil; 2 Laboratório de Estudos Herpetológicos e Paleoherpetológicos, Departamento de Biologia, Universidade Federal Rural de Pernambuco, Recife, Brazil; 3 Colección Nacional de Arácnidos, Departamento de Zoología, Instituto de Biología, Universidad Nacional Autónoma de México, Ciudad de México, México; 4 Department of Earth and Environmental Sciences, University of Milano-Bicocca, Milan, Italy; 5 Programa de Pós-graduação em Biodiversidade, Centro de Ciências Agrárias, Universidade Federal da Paraíba, Areia, Brazil; 6 Programa de Pós-graduação em Biociência Animal, Departamento de Morfologia e Fisiologia Animal, Universidade Federal Rural de Pernambuco, Recife, Brazil; University of Windsor, CANADA

## Abstract

Acoustic signals play a fundamental role in the lives of anurans. With the increasing prevalence of wind farms in Caatinga ecosystems, our goal was to assess whether the noise generated by this energy source poses a threat to anuran communication. We analyzed acoustic parameters of the advertisement calls from 181 adult males, of the species *Scinax pachycrus*, *Scinax x-signatus*, and *Physalaemus cicada*. Call activity was recorded across noise gradients in 19 temporary ponds with similar vegetation structures, distributed in two wind farms. Our results revealed a significant relationship between wind turbine noise and changes in acoustic parameters of the three species, distinctly influencing their spectral and temporal parameters of the advertisement calls. Dominant frequency, frequency range of *Scinax pachycrus* were affected by the noise, decreasing in noisier temporary ponds, while *Scinax x-signatus* decreased call amplitude and call pulses. On the other hand, *Physalaemus cicada* showed changes only in temporal variables, with reductions in call duration and call pulses, and an increase in call rate to cope whit noise in temporary ponds. Therefore, noise pollution becomes particularly concerning for the anurans of the Caatinga, as the areas of greatest interest for wind power generation overlap with priority areas for biodiversity conservation.

Acoustic signaling stands out as a prominent trait in anurans, serving as their primary mode of communication and playing an essential role in the reproduction and social organization of populations [1]. These signals convey valuable information, including body size and reproductive fitness, crucial for the reproduction and survival of various anuran species [2, 3]. Therefore, it is imperative that signals effectively convey the intended message to the target audience. In noisy environments, species often attempt to adjust the acoustic structure of their signals to enhance the signal-to-noise ratio [4]. This adjustment can involve short-term changes related to signal plasticity, such as alterations in amplitude, temporal features, or frequency [5]. Alternatively, it may involve long-term evolutionary changes, as suggested by the Acoustic Adaptation Hypothesis [2]. The Acoustic Adaptation Hypothesis posits that the environment in which acoustic communication occurs should favor vocalization traits that minimize attenuation and signal degradation [6]. While animals like insects and frogs have natural adaptations to cope with ambient noise [7], an increase in noise, especially from a new and different source like wind farms, represents a novel stimulus that alters the acoustic conditions of natural habitats [8,9].

Wind energy is recognized as a competitive, reliable, and renewable technology [10], offering an alternative for reducing greenhouse gas emissions and facilitating the transition to a low-carbon economy for nations [11]. However, its implementation is associated with challenges, including the suppression of vegetation, soil exposure and erosion, and the creation of new roads and transmission lines [10]. Furthermore, some studies have linked wind farm noise to various impacts on animals. These investigations have reported alterations in bird vocalization, such as the suppression of territorial calls [12] and changes in calling parameters [13]. In mammals, deleterious effects include changes in anti-predator behavior in squirrels [14] and chronic stress in badgers [15]. Concerning anurans, the impact of wind farm noise encompasses behavioral changes in call activity [16], reduced species richness [17], and immune system suppression [18]. The recent implementation of wind farms in Caatinga ecosystems has led to significant landscape changes, such as the suppression of native vegetation, soil waterproofing (due to soil compaction), construction of transmission lines, and an increase in noise pollution [19].

The Caatinga, a unique seasonally tropical dry forest exclusive to Brazil, is a region of significant interest for the expansion of wind farms [20,21]. Moreover, areas designated for existing or potential wind farm installations coincide with crucial biodiversity conservation zones [21]. The Caatinga is estimated to have a power generation capacity of 75GW, constituting 70% of the total priority areas for wind power generation in Brazil [22]. Given that noise can impair sound perception and information exchange, wind farm noise emerges as a potential threat with the capacity to negatively impact various animal groups, warranting increased attention [8,9,23]. This is particularly relevant for species like anurans, whose reproduction and survival (e.g., mating choice, predator detection, and territory defense) depend on the accurate emission and perception of environmental signals and cues [7,24]. Therefore, changes in the Caatinga soundscape caused by wind farm noise could have adverse effects on anurans, necessitating urgent research.

Taking this into account, our study aims to assess behavioral changes in the advertisement calls of three anuran species exposed to wind farm noise in the Caatinga, Northeastern Brazil. Given that noise pollution can affect anuran calling behavior in various ways [9], we hypothesize that wind turbine noise could alter spectral and temporal call parameters of the species. Additionally, considering evidence that substrate-borne noise can negatively impacting calling behavior in anurans [16], we also expect that calling animals partially immersed in temporary ponds would be more affected by the noise.

## Methods

### Field data sampling

Most Caatinga anurans, including the three species under investigation, breed during the short rainy season from April to August [19]. We recorded the advertisement calls of these species throughout the rainy season of 2019 in Caetés municipality, Pernambuco State, Brazil. Our field study was conducted in two wind farms located in Ventos de Santa Brígida Parks, which collectively house 107 turbines. The turbines are of the GE Energy 1.7-100 model, possessing a power generating capacity of 1,700 KW. These turbines, standing at a height of 96 m, consist of three propellers, each 52 m in length. The vegetation in the study area has undergone alterations due to human-made actions, characterized by shrub and tree-shrub formations [19].

### Wind farm noise recording

We carefully selected nine temporary ponds within each wind farm to capture the aerodynamic noise resulting from the interaction between the blades and the wind. The chosen ponds shared similar environmental characteristics, including an average diameter of 42 m², and were situated within open shrub areas. We also identified a temporary pond located 2 km away from the wind farm sites, possessing environmental characteristics similar to other 18 ponds, that is outside the context of noise from wind farms and which we call the less noisy area. Wind farm noise was recorded at 16:00 pm using a decibel meter (Instrutemp ITDEC 4000, 0.1 dB accuracy, weighting curve C) at ground level for 60 seconds. The recordings were conducted in the 19 temporary ponds, establishing a noise gradient at each wind farm during the rainy season in March 2019. Subsequently, we categorized the remaining values into three noise classes based on the values observed across all sampled ponds - hereafter referred to as noise classes (S1 Table). In addition, S1 Table shows the range and average noise per class expressed in decibels, as well as the range and average distance between temporary ponds and wind farms expressed in meters.

### Focal species

**Fig 1:**
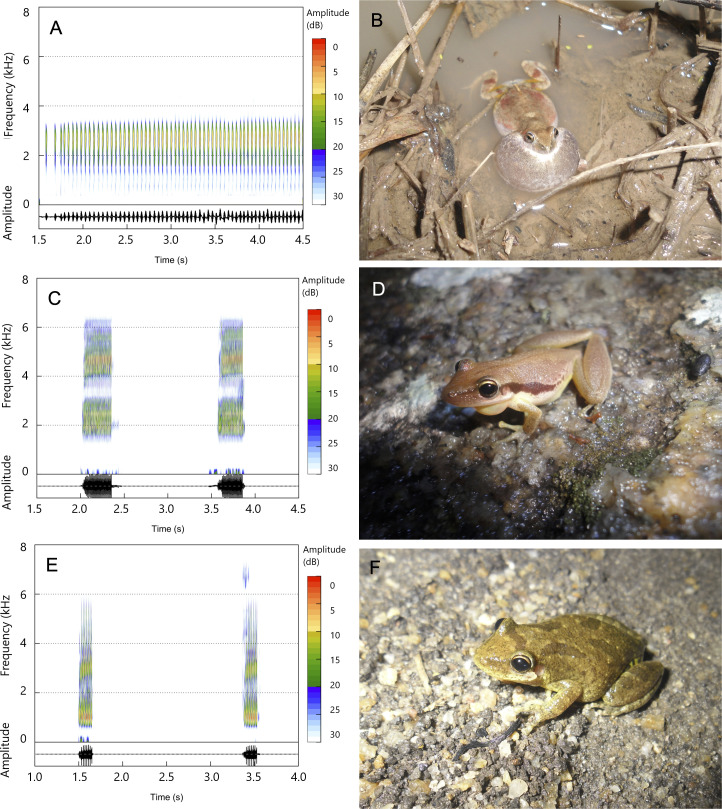
Oscillogram (bottom) and sound spectrogram (top) of *Physalaemus cicada* (A, B), *Scinax pachycrus* (C, D), and *Scinax x-signatus* (E, F).

During our field experimental study, we recorded the advertisement calls of 181 individual adult males, representing the Pocao Snouted Treefrog (*Scinax pachycrus*, n = 69), Venezuela Snouted Treefrog (*Scinax x-signatus*, n = 69), and Maracas Dwarf Frog (*Physalaemus cicada*, n = 43). These individuals were equally distributed among the 19 sampled temporary ponds. We selected these three species because they are commonly found in the study area [19] and exhibit distinct acoustic profiles (Fig 1).

*Physalaemus cicada* is a small-sized terrestrial leptodactylid (Snout–to–vent–length–SVL 27 mm), vocalizing partially immersed in temporary ponds (Fig 1A-B). The advertisement call of *P. cicada* is composed of a continuous and multipulsed note, with a duration between 0.004-0.047 seconds, a call rate of 1,500 notes per minute, and a dominant frequency between 1.4-3.5 kHz [25].

*Scinax pachycrus* (Fig 1C-D) and *S. x-signatus* (Fig 1E-F) are small-sized, semi-arboreal hylids (Snout–to–vent–length–SVL 30 mm and 35 mm, respectively) which vocalize at the edge of temporary ponds. The advertisement call of *S. pachycrus* consists of one multipulsed note, with a duration between 0.2-0.3 seconds, a call rate ranging from 28-51 notes per minute, and a dominant frequency between 1.6-4.4 kHz (present study). The advertisement call of *S. x-signatus* is composed of one multipulsed note, with a duration between 0.14-0.19 seconds, a call rate ranging from 45-66 notes per minute, and a dominant frequency between 0.9-1 kHz [26].

To record the advertisement calls, we used a YOGA HT81 unidirectional microphone coupled to a Tascam DR40 digital recorder, configured at 44.1 kHz, with a 16-bit resolution. We standardized the recording by positioning the microphone at 50 cm from the animals engaged in calling activity while maintaining a stable recorder gain.

### Acoustic data

We analyzed acoustic parameters of the advertisement calls using Raven Pro 1.5 software [27]. Spectral parameters included Dominant Frequency (the frequency containing the highest calling energy measured in Hertz), Frequency Range (defined as the result of subtracting the 10% frequency from the 90% frequency in Raven software measured in Hertz), and Call Amplitude (standardized measures of calling intensity in decibels). Temporal parameters included Call Duration (the duration of a call in seconds), Call Pulses (the number of pulses repeated within a call), and Call Rate (the number of calls emitted per minute). All parameter definitions, except Frequency Range, followed [28]; for more details, see S2 Table. Temporal parameters were measured from oscillograms, while spectral parameters were measured from spectrograms created using a Hann window, a window length FFT of 512 points, and 50% overlap. Spectrogram settings were Hann, window size =  1024 samples, 3 dB bandwidth =  270 Hz, Overlap =  85%, hop size =  0.792, DFT size =  1024 samples, and grid spacing =  46.9 Hz. All other settings followed the ‘default’ of Raven.

### Data analysis

In our data analysis, we employed the ‘car’ package to conduct a Multivariate Analysis of Covariance (MANCOVA), investigating potential changes in the vocal parameters of the three species in response to wind turbine noise. The acoustic parameters of each species were transformed using the Box-Cox technique (‘MASS’ package) to ensure normality and homogeneity of the data and covariance matrices [29].

In the subsequent stage of MANCOVA construction, the Noise variable was designated as a factor variable. To validate the assumptions of the MANCOVA model, we conducted multivariate and univariate normality analyses. For multivariate normality, we used the Henze-Zirkler test with the ‘MVN’ package [30]. Then, to assess the homogeneity of covariance matrices among groups defined by the Noise factor, we used Box’s M test with the boxM() function from the ‘car’ package [31]. Finally, we checked univariate normality using Shapiro-Wilk tests, employing the shapiro.test() function from the base R package. Additionally, we evaluated univariate homogeneity through the Levene test, using the leveneTest() function from the ‘car’ package.

Furthermore, we used Pillai’s test values to assess the significance of main effects and interactions, based on variation from 0 to 1, where values close to 0 indicate limited association between groups defined by independent factors and values close to 1 indicate strong association (S3 Table). We observed significant p-values from MANCOVA, indicating statistically significant differences among groups defined by independent factors. To visualize the relationships of each acoustic parameter with the noise gradient, we constructed boxplots using the ‘ggplot2’ package [32]. All statistical analyses and figures were conducted in the R software [33].

### Ethical Note

We recorded the advertisement call of 181 individual males of the species Pocao Snouted Treefrog, Venezuela Snouted Treefrog, and Maracas Dwarf Frog, all included in Last Concern category of Red List of Threatened Species from International Union for Conservation of Nature [34]. We conducted the study in compliance with the Comitê de Ética e Uso Animal from Universidade Federal Rural de Pernambuco (CEUA-UFRPE, permit #144/2019) guidelines for animals use in experimentation. All the methods were performed in compliance with the Brazilian laws on animal research under permission of the Sistema de Autorização e Informação em Biodiversidade (SISBio, permit #67297-1/2019). To minimize distress, pain or the welfare impact on animals engaged in calling activity at the sites, we did not collect the individuals.

## Results

Our results revealed a significant relationship between wind turbine noise and changes in acoustic parameters of the three species (S2 Table). Based on the MANCOVA analysis, we observed a significant relationship between wind turbine noise and distinct acoustic parameters of *Scinax pachycrus* (Pillai =  0.78; F =  3.67; p < 0.01), *Scinax x-signatus* (Pillai =  0.68; F =  3.07; p < 0.01), and *Physolaemus cicada* (Pillai =  0.74; F =  1.98; p < 0.01).

**Fig 2:**
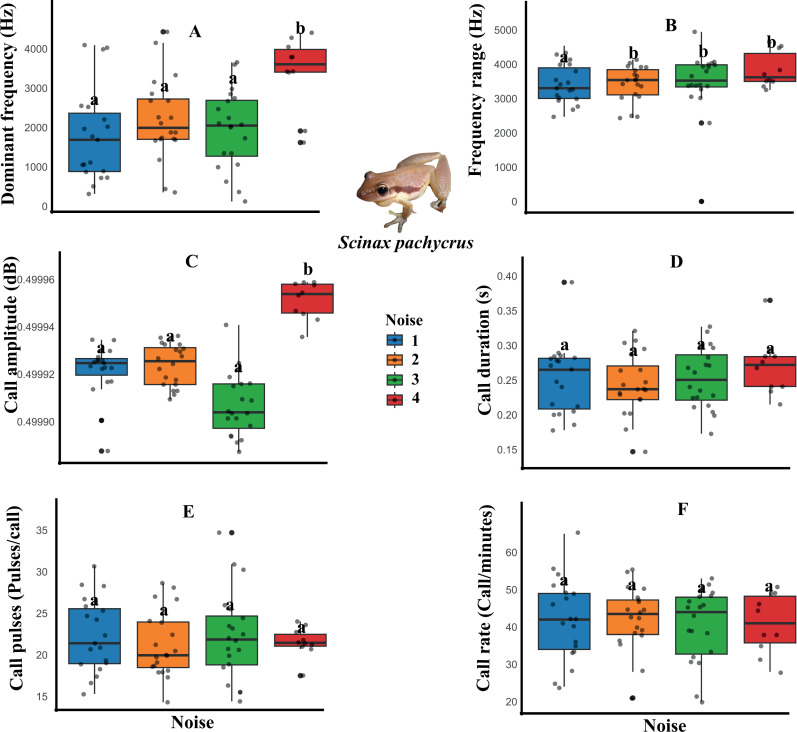
Boxplot of the acoustic parameters of *Scinax pachycrus* in response to wind farm noise. The graphs show that calling males found in the ponds of the most intense noise classes had a reduction in the parameters dominant frequency (A), frequency range (B) and call amplitude (C). * Letters above the bars indicate significant differences between the noise categories.

**Fig 3:**
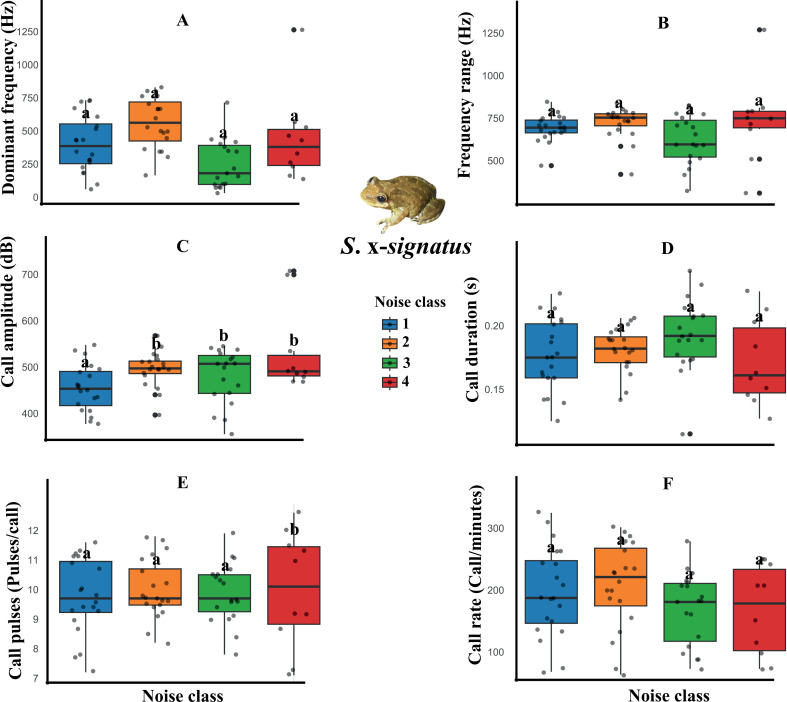
Boxplot of the acoustic parameters of *Scinax x-signatus* in response to wind farm noise. The graphs show that calling males found in the ponds of the most intense noise classes had a reduction in the parameters call amplitude (C) and call pulses (E). * Letters above the bars indicate significant differences between the noise categories.

**Fig 4:**
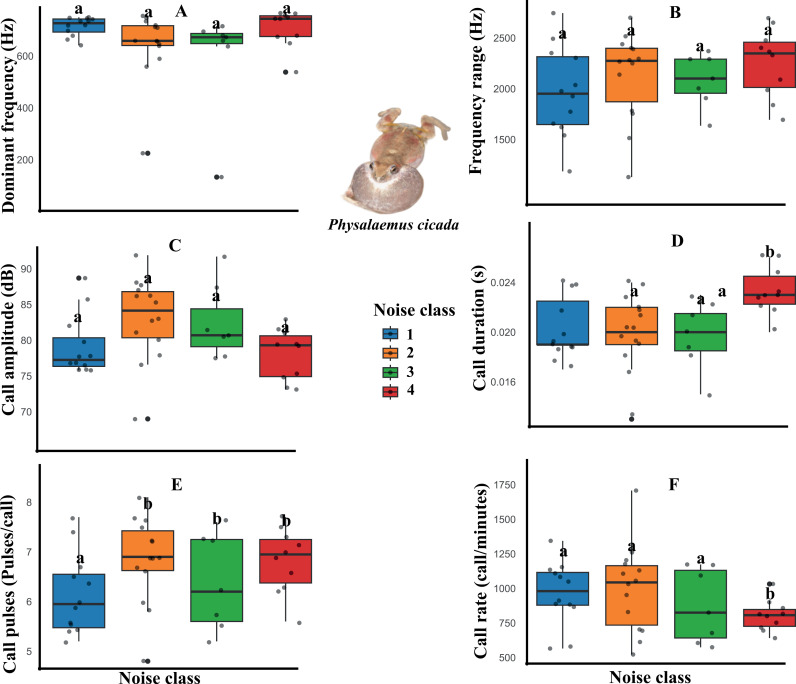
Boxplot of the acoustic parameters of *Physalaemus cicada* in response to wind farm noise. The graphs show that calling males found in the ponds of the most intense noise classes had a reduction in the parameters call duration (D), call pulses (E) and an increase in call rate (F). *  Letters above the bars indicate significant differences between the noise categories.

However, wind turbine noise distinctly influenced the spectral and temporal parameters of the advertisement calls of the three species. For example, the dominant frequency, frequency range, and call amplitude variables of *Scinax pachycrus* were affected by the noise, decreasing in noisier temporary ponds (Fig 2, Table 1). Regarding the call of *Scinax x-signatus*, decreases were observed in the call amplitude and call pulses variables (Fig 3, Table 1). On the other hand, *Physalaemus cicada* showed changes only in temporal variables of the call, with decreases in call duration and call pulses, and increases in call rate in noisier temporary ponds (Fig 4, Table 1).

**Table 1:** Summary of results based on the results of Mancova’s analysis, highlighting the relationship between the acoustic parameters of the three studied species and the noise gradient recorded in two wind farms and a low-noise area. “n.s.” denotes non-significant changes.

Anuran species	Acoustic variables					
	Dominant frequency	Call amplitude	Frequency range	Call pulses	Call duration	Call rate
*Scinax pachycrus*	Decreased	Decreased	Decreased	n.s.	n.s.	n.s.
*Scinax x-signatus*	n.s.	Decreased	n.s.	Decreased	n.s.	n.s.
*Physalaemus cicada*	n.s.	n.s.	n.s.	Decreased	Decreased	Increased

## Discussion

The results of our field study aimed to assess the effect of wind farm noise on the calling behavior of three anuran species from the Caatinga dry forests. Our findings suggest a significant relationship between wind farm noise and changes in both spectral and temporal call parameters, supporting our initial expectations. Furthermore, our assumption that *Physalaemus cicada* would be the species with the most acoustic parameters affected by noise was supported since it had all temporal parameters altered with noise increase. *Scinax pachycrus* also had all its spectral parameters significantly affected by noise, highlighting the potential of wind farm noise to affect the calling behavior of anurans in different ways.

### Wind farm noise effects on calling spectral parameters

The dominant frequency is the frequency containing the highest energy and is a biologically relevant acoustic parameter, as the most perceived frequency of the signal [8,23,35]. It’s essential for conspecific recognition, playing a crucial role in anuran sexual selection [24]. In this study, *S. pachycrus* showed a decrease in the dominant frequency of the call in noisier ponds. However, our findings are not surprising, as a recent review has shown decreases in dominant frequency for two anuran species exposed to human-made noise, *Boana bischoffi*, and *Pelophylax ridibundus* (while ten species increased, and eight did not change dominant frequency; see [9]. These authors suggest that the reduction of dominant frequency could be an efficient strategy for some species under conditions of stress caused by noise, favored by the longer-distance propagation of low-pitched signals. On the other hand, the decrease in dominant frequency may lead *S. pachycrus* to compete with heterospecific males for frequency bands, generating interspecific competition [7,36].

Dominant frequency is inversely correlated with the body size of individuals and their sound production structures, being associated with reproductive performance and survival [2]. Females of many anuran species prefer low-frequency conspecific calls, as it reflects the reproductive fitness of males, favoring larger individuals [36]. On one hand, noisy ponds may alter the ability of females to distinguish the origin of signals emitted by males, reducing the chances of attracting conspecific mates [37]. On the other hand, our findings indicate that elevated levels of wind farm noise lead *S. pachycrus* to produce calls with a lower dominant frequency, which could become an advantage for larger males in attracting females, as it is favored by the longer-distance propagation of low-frequency signals [38]. Furthermore, decreases in dominant frequency in *S. pachycrus* associated with the increase in wind farm noise challenge the hypothesis that acoustic communication in species with spectral overlap between signal and noise would be most negatively affected. Since in *S. pachycrus* the dominant frequency is emitted between 1.6-4.4 kHz (present study), there is no spectral overlap with wind farm noise, which is emitted at 0.4 kHz. This finding demonstrates that human-made noise can alter the calling behavior of anurans even when emitted at low frequencies and with small spectral overlap [39].

Changes in call amplitude were observed in *Scinax pachycrus* and *S. x-signatus*, with both species decreasing this acoustic parameter in noisier temporary ponds. This decrease contradicts the strategy commonly used by animals in noisy environments, where call amplitude is typically raised in response to noise, consistent with the Lombard effect [40]. Indeed, previous studies with anurans have shown increased call amplitude in *Kurixalus chaseni* [41] and *Scinax nasicus* [42] in noisy conditions. On the other hand, other species have shown a decrease in call amplitude [38]. Similarly to our findings for *S. pachycrus*, [43] found that *Pelophylax ridibundus* reduced both amplitude and frequency of the call when exposed to noise. Previous work on frog calling energetics indicates that calling is likely the most energetically expensive activity for a male [24,44]. Therefore, to reduce energy consumption while maximizing signal transmission [1], the species might lower call amplitude and dominant frequency, favoring long-distance propagation of low-pitched signals [45]. Meanwhile, species like *S. x-signauts*, when reducing call amplitude and call pulses, might be saving energy.

Our findings also revealed that frequency range of *Scinax pachycrus* call decreased in noisy temporary ponds. Frequency modulation is a response to noise pollution that increases the proportion of the signal that is free from acoustic masking [8,23,35]. In animals such as anurans, frequency depends on body size, and higher frequency calls may advertise small size and low fitness [36,46]. Although we did not evaluate the effect of noise on the minimum call frequency of *S. pachycrus*, bird studies suggest that changes in this parameter could bring disadvantages to the species. For example, a playback study with male cardinals *Cardinalis cardinalis* with higher frequency calls elicited weaker territorial responses from members of the same species, showing that minimum frequency adjustments require more energy in defending the territory [47]. Consequently, there is increasing stress, predisposition to mortality and reducing the reproductive success of the species [48]. However, it is not clear how changes in the frequency range caused by noise can affect anurans.

### Wind farm noise effects on calling temporal parameters

In the present study, *P. cicada* emitted calls of significantly shorter duration towards temporary ponds with high noise levels. Anuran males exhibit diverse responses when confronted with human-made noise, being capable of minimizing acoustic masking by transmitting more information per call and increasing redundancy when parts of the signal are lost [8]. Its known that males of some anuran species do not increase their call effort in the presence of high-intensity noise because the energy expenditure might not compensate for the increased effort [9]. In addition, previous studies with *Hyla arborea* [49] and *Boana leptolineata* [50], using traffic noise stimuli, demonstrated that species decreased call duration as the noise increased, corroborating our findings for *P. cicada*. However, evidence suggests that females of some anuran species have a preference for males that emit long-lasting calls [51]. Therefore, males are faced with a trade-off between the energetic cost of calls and the maintenance of attractiveness of mate in noisy ponds [38], a topic that requires further studies.

We also found that *P. cicada* and *S. x-signatus* emitted calls with fewer pulses in noisier temporary ponds. Evidence suggests that even subtle reductions in just a few pulses within a call can influence the call preference of anuran females [51]. On the other hand, [52] showed that females of the same species (*Hila versicolor*) did not show differences in the attractiveness of calls with varied pulses (9-27 pulses) in the presence of noise stimuli. However, these authors suggest that pulses per call explained just a small portion of the total variation in male success. Although biological consequences of the reduction in call pulses are not clear enough, it becomes necessary to understand its influence on the mate choice by females in noisy environments, and consequently, on the reproductive success of the species.

Unlike all other acoustic parameters analyzed here, our results showed that the call rate of *P. cicada* increased in noisier temporary ponds. In anurans, reproductive success is related to vocalization effort; since it is an energetically costly activity increases in call rate have direct consequences on the fitness and survival rate of the species [4,24,53]. Thus, signal redundancy is a common response in animals that increases the probability of sound detection in the presence of noise interference [35]. In this way, *P. cicada* alters the temporal characteristics of their calls so that they are not masked by background noise, minimizing attenuation of the acoustic signal [9,38]. Nonetheless, remains unclear whether the alterations in calling characteristics compensate for possible effects of wind farm noise on breeding success [38].

### Wind farm noise effects on population and community levels

Assessing the impact of wind farm noise on anuran population and community levels is challenging because species seem to respond to the noise in different ways. This helps us understand why most studies on the effects of human-made noise on animals are primarily focused on the individual level. When exposed to wind farm noise [17,18] or substrate-borne vibrations emitted by them [16] the species may alter call parameters. Although wind farm noise has been linked to increases in corticosterone levels and reductions in the immune system in anuran species like *Dryophytes japonicus* [18], no negative effects were detected on species richness and diversity patterns in the same anuran community sampled in our study [19].

In the anuran reproduction, mating choice is determined by females [24]. Therefore, in the presence of human-made noise females may become less selective, shifting from discriminating among available male vocalizations to simply locating a male [9]. This is especially relevant for anurans from the Caatinga, where around 90% of the species are habitat generalists and exhibit explosive-type breeding [among a universe of twenty sampled species, 19]. In this highly seasonal ecosystem, the short rainy season limits the time and sites for anuran reproduction, providing few opportunities for females to choose partners with better reproductive fitness while dealing with wind farm noise. However, these hypotheses need further testing. Finally, future assessments of the negative impacts of wind farms should include a higher number of samples sites and employ multiple methods of data collection to understand the noise effects on biologically crucial aspects such as reproductive fitness, fecundity, reproductive success, tadpoles, hormones, and morphology [19].

## Conclusions

This experimental field study represents the first attempt to assess the impact of noise pollution from wind farms on anuran communication in the Caatinga dry forests. Our findings establish a significant correlation between wind farm noise and alterations in both spectral and temporal parameters of calls in three anuran species prevalent in these semi-arid ecosystems. Although the species studied here are in the IUCN Least Concern category, 41% of amphibians are globally threatened [34]. Considering the pivotal role of acoustic communication in reproduction, territory defense, and predator detection [1,4], wind farm noise may impede sound perception and compromise information exchange, especially for anurans with some degree of threat and endemic species.

From a conservation standpoint, this issue is particularly alarming in Caatinga ecosystems, where areas of substantial interest for wind power generation coincide with priority zones for biodiversity conservation [21]. In this way, we emphasize that efforts to mitigate the effects of wind farm noise in Caatinga should focus on suitable spatial planning and technological innovations, such as quieter wind turbine propellers, which would likely be more effective than traditional noise barriers (e.g., those used on highways). Additionally, mitigation strategies should align with biologically sensitive periods of the species, such as the short breeding season of Caatinga anurans [19].

## Supporting informtion

S1 Table Noise classes and the distance of temporary ponds to the two wind farms in Caetés, Pernambuco State, Brazil. For both wind farms, each class is represented by three temporary ponds. The table shows the range and average noise level per class, expressed in decibels, as well as the range and average distance between temporary ponds and wind farms, expressed in meters. Although not shown in the table below, the environmental noise in the control area was 67.7 dB. (DOCX)

S2 Table Noise classes in temporary ponds distributed in two wind farms in Caetés, Pernambuco State, Brazil. For both wind farms, each class was represented by three temporary ponds. The table displays the range and average noise per class expressed in decibels with frequency weighting C. Additionally, we provided a summary of the data set used in MANCOVA analysis, including sites, noise classes, and sampled species. Ranges, averages, and standard deviations are presented for spectral variables (Dominant Frequency, Frequency Range, and Call Amplitude), temporal variables (Call duration, Call Pulses, and Call Rate). (DOCX)

S3 Table Pillai’s test with significance of the relationship between wind turbine noise and advertisement call parameters of the species with their respective *F-values*, as well as the *P-value* of the MANCOVA. (DOCX)

## References

[1] Wells KD, Schwartz JJ. The behavioral ecology of anuran communication. In: Narins PM, Feng AS, Fay RR, Popper AN. Editors. Hearing and Sound Communication in Amphibians (pp. 44–86). New York, NY: Springer New York. doi: 10.1007/978-0-387-47796-1_3

[2] Ey E, Fischer J. The “acoustic adaptation hypothesis”—a review of the evidence from birds, anurans and mammals. Bioacoustics. 2009;19(1–2):21–48. doi: 10.1080/09524622.2009.9753613

[3] Chen Z, Wiens JJ. The origins of acoustic communication in vertebrates. Nat Commun. 2020;11(1):369. doi: 10.1038/s41467-020-14356-3 31953401 PMC6969000

[4] Endler JA. Signals, signal conditions, and the direction of evolution. The American Naturalist. 1992;139:S125–53. doi: 10.1086/285308

[5] Brumm H, Zollinger S. Avian vocal production in noise. In: Brumm H. Editors. Animal Communication and Noise. Animal Signals and Communication, vol 2. Springer, Berlin, Heidelberg; 2013. doi: 10.1007/978-3-642-41494-7_7

[6] Morton ES. Ecological sources of selection on avian sounds. The American Naturalist. 1975;109(965):17–34. doi: 10.1086/282971

[7] Gerhardt HC, Huber F. (2002). Acoustic communication in insects and anurans: common problems and diverse solutions. Chicago: University of Chicago Press. doi: 10.1121/1.1591773

[8] Duquette CA, Loss SR, Hovick TJ. A meta‐analysis of the influence of anthropogenic noise on terrestrial wildlife communication strategies. Journal of Applied Ecology. 2021;58(6):1112–21. doi: 10.1111/1365-2664.13880

[9] Zaffaroni-Caorsi V, Both C, Márquez R, Llusia D, Narins P, Debon M, et al. Effects of anthropogenic noise on anuran amphibians. Bioacoustics. 2022;32(1):90–120. doi: 10.1080/09524622.2022.2070543

[10] Dai K, Bergot A, Liang C, Xiang W-N, Huang Z. Environmental issues associated with wind energy – A review. Renewable Energy. 2015;75:911–21. doi: 10.1016/j.renene.2014.10.074

[11] Gasparatos A, Doll CNH, Esteban M, Ahmed A, Olang TA. Renewable energy and biodiversity: Implications for transitioning to a Green Economy. Renewable and Sustainable Energy Reviews. 2017;70:161–84. doi: 10.1016/j.rser.2016.08.030

[12] Zwart MC, Dunn JC, McGowan PJK, Whittingham MJ. Wind farm noise suppresses territorial defense behavior in a songbird. BEHECO. 2015;27(1):101–8. doi: 10.1093/beheco/arv128

[13] Whalen CE, Brown MB, McGee J, Powell LA, Walsh EJ. Effects of wind turbine noise on the surrounding soundscape in the context of greater-prairie chicken courtship vocalizations. Applied Acoustics. 2019;153:132–9. doi: 10.1016/j.apacoust.2019.04.022

[14] Rabin LA, Coss RG, Owings DH. The effects of wind turbines on antipredator behavior in California ground squirrels (Spermophilus beecheyi). Biological Conservation. 2006;131(3):410–20. doi: 10.1016/j.biocon.2006.02.016

[15] Agnew RCN, Smith VJ, Fowkes RC. Wind turbines cause chronic stress in badgers (meles meles) in great britain. J Wildl Dis. 2016;52(3):459–67. doi: 10.7589/2015-09-231 27187031

[16] Caorsi V, Guerra V, Furtado R, Llusia D, Miron LR, Borges-Martins M, et al. Anthropogenic substrate-borne vibrations impact anuran calling. Sci Rep. 2019;9(1):19456. doi: 10.1038/s41598-019-55639-0 31857629 PMC6923410

[17] Trowbridge CM, Litzgus JD. Wind farms alter amphibian community diversity and chorusing behavior. Herpetologica. 2022;78(2):. doi: 10.1655/herpetologica-d-21-00032

[18] Park J-K, Do Y. Wind turbine noise behaviorally and physiologically changes male frogs. Biology (Basel). 2022;11(4):516. doi: 10.3390/biology11040516 35453715 PMC9031316

[19] de Oliveira RF, de Araujo Lira AF, Zaffaroni-Caorsi V, de Moura GJB. Wind farm noise and anuran diversity patterns: a case study in Brazilian seasonal dry tropical forest. Bioacoustics. 2023;32(5):544–55. doi: 10.1080/09524622.2023.2204325

[20] Bernard E, Paese A, Machado RB, Aguiar LM de S. Blown in the wind: bats and wind farms in Brazil. Natureza & Conservação. 2014;12(2):106–11. doi: 10.1016/j.ncon.2014.08.005

[21] Neri M, Jameli D, Bernard E, Melo FPL. Green versus green? Adverting potential conflicts between wind power generation and biodiversity conservation in Brazil. Perspectives in Ecology and Conservation. 2019;17(3):131–5. doi: 10.1016/j.pecon.2019.08.004

[22] Agência Nacional de Energia Elétrica. Atlas de Energia Elétrica do Brasil. Technical report, 3^a^ edição, Brasília; 2008, p. 236.

[23] Shannon G, McKenna MF, Angeloni LM, Crooks KR, Fristrup KM, Brown E, et al. A synthesis of two decades of research documenting the effects of noise on wildlife. Biol Rev Camb Philos Soc. 2016;91(4):982–1005. doi: 10.1111/brv.12207 26118691

[24] Wells KD. The ecology and behavior of amphibians. University of Chicago press; 2007. doi: 10.7208/9780226893334

[25] Hepp F, Pombal JPJ. Review of bioacoustical traits in the genus *Physalaemus* Fitzinger, 1826 (Anura: Leptodactylidae: Leiuperinae). Zootaxa. 2020;4725(1):zootaxa.4725.1.1. doi: 10.11646/zootaxa.4725.1.1 32230594

[26] Araujo-Vieira K, Pombal Jr. JP, Caramaschi U, Novaes-e-Fagundes G, Orrico VGD, Faivovich J. A neotype for *Hyla x-signata* Spix, 1824 (Amphibia, Anura, Hylidae). Pap Avulsos Zool. 2020;60:e20206056. doi: 10.11606/1807-0205/2020.60.56

[27] The Cornell Lab of Ornithology. Raven Pro: Interactive Sound Analysis Software (Version 1.6.4) [Computer software]. Ithaca, NY; 2012. Available from: https://ravensoundsoftware.com/

[28] Köhler J, Jansen M, Rodríguez A, Kok PJR, Toledo LF, Emmrich M, et al. The use of bioacoustics in anuran taxonomy: theory, terminology, methods and recommendations for best practice. Zootaxa. 2017;4251(1):1–124. doi: 10.11646/zootaxa.4251.1.1 28609991

[29] Venables WN, Ripley BD. Modern Applied Statistics with S, Fourth edition. Springer, New York; 2002.

[30] Korkmaz S, Goksuluk D, Zararsiz G. Mvn: An r package for assessing multivariate normality. The R Journal. 2014;6(2):151–62.

[31] Fox J, Weisberg S. An R Companion to Applied Regression, Third edition. Sage, Thousand Oaks CA; 2019.

[32] Wickham H. ggplot2: Elegant Graphics for Data Analysis. Springer-Verlag, New York; 2016.

[33] R Core Team. R: A language and environment for statistical computing. 2021. Available from: https://www.r-project.org/

[34] International Union for Conservation of Nature. Red List of Threatened Species. 2023. Available from: https://www.iucnredlist.org

[35] Simmons AM, Narins PM. Effects of anthropogenic noise on amphibians and reptiles. In: Slabbekoorn H., Dooling R., Popper. A., & Fay R. (editors). Effects of anthropogenic noise on animals. 1st ed. Berlin, Germany: Springer-Verlag; 2018. p. 179–208.

[36] Gerhardt HC. Selective responsiveness to long-range acoustic signals in insects and anurans. Am Zool. 1994;34(6):706–14. doi: 10.1093/icb/34.6.706

[37] Bee MA, Swanson EM. Auditory masking of anuran advertisement calls by road traffic noise. Animal Behaviour. 2007;74(6):1765–76. doi: 10.1016/j.anbehav.2007.03.019

[38] Cunnington GM, Fahrig L. Plasticity in the vocalizations of anurans in response to traffic noise. Acta Oecologica. 2010;36(5):463–70. doi: 10.1016/j.actao.2010.06.002

[39] Tamura H, Ohgami N, Yajima I, Iida M, Ohgami K, Fujii N, et al. Chronic exposure to low frequency noise at moderate levels causes impaired balance in mice. PLoS One. 2012;7(6):e39807. doi: 10.1371/journal.pone.0039807 22768129 PMC3387207

[40] Brumm H, Zollinger SA. The evolution of the Lombard effect: 100 years of psychoacoustic research. Behav. 2011;148(11–13):1173–98. doi: 10.1163/000579511x605759

[41] Yi YZ, Sheridan JA. Effects of traffic noise on vocalisations of the rhacophorid tree frog *Kurixalus chaseni* (Anura: Rhacophoridae) in Borneo. Raffles Bulletin of Zoology. 2019;67:77–82.

[42] Leon E, Peltzer PM, Lorenzon R, Lajmanovich RC, Beltzer AH. Effect of traffic noise on *Scinax nasicus* advertisement call (Amphibia, Anura). Iheringia, Sér Zool. 2019;109:e2019007. doi: 10.1590/1678-4766e2019007

[43] Lukanov S, Simenovska-Nikolova D, Tzankov N. Effects of traffic noise on the locomotion activity and vocalization of the marsh frog, *Pelophylax ridibundus*. North West Journal of Zoology. 2014;10(2):359–64.

[44] Pough FH, Magnusson WE, Ryan MJ, Wells KD, Taigen TL. 1992. In: Feder ME, Burggren WW Editors, Environ Physiol Amphib 1st ed. Chicago (IL): University of Chicago Press; pp. 395–436.

[45] Forrest TG. From sender to receiver: propagation and environmental effects on acoustic signals. Am Zool. 1994;34(6):644–54. doi: 10.1093/icb/34.6.644

[46] Brumm H. The impact of environmental noise on song amplitude in a territorial bird. Journal of Animal Ecology. 2004;73(3):434–40. doi: 10.1111/j.0021-8790.2004.00814.x

[47] Luther D, Magnotti J. Can animals detect differences in vocalizations adjusted for anthropogenic noise?. Animal Behaviour. 2014;92:111–6. doi: 10.1016/j.anbehav.2014.03.033

[48] Injaian AS, Poon LY, Patricelli GL. Effects of experimental anthropogenic noise on avian settlement patterns and reproductive success. Behavioral Ecology. 2018;29(5):1181–9. doi: 10.1093/beheco/ary097

[49] Lengagne T. Traffic noise affects communication behaviour in a breeding anuran, *Hyla arborea*. Biological Conservation. 2008;141(8):2023–31. doi: 10.1016/j.biocon.2008.05.017

[50] Caorsi VZ, Both C, Cechin S, Antunes R, Borges-Martins M. Effects of traffic noise on the calling behavior of two Neotropical hylid frogs. PLoS One. 2017;12(8):e0183342. doi: 10.1371/journal.pone.0183342 28854253 PMC5576727

[51] Gerhardt HC, Tanner SD, Corrigan CM, Walton HC. Female preference functions based on call duration in the gray tree frog (*Hyla versicolor*). Behavioral Ecology. 2000;11(6):663–9. doi: 10.1093/beheco/11.6.663

[52] Schwartz JJ, Buchanan BW, Gerhardt HC. Female mate choice in the gray treefrog (*Hyla versicolor*) in three experimental environments. Behavioral Ecology and Sociobiology. 2001;49(6):443–55. doi: 10.1007/s002650100317

[53] Brepson L, Voituron Y, Lengagne T. Condition-dependent ways to manage acoustic signals under energetic constraint in a tree frog. Behavioral Ecology. 2012;24(2):488–96. doi: 10.1093/beheco/ars189

