# Supplementary material for: Wind farm noise negatively impacts the calling behavior of three frogs in Caatinga dry forests
Source: PLoS One. 2025 Mar 19;20(3):e0318517. doi: 10.1371/journal.pone.0318517 (PMC11922283; doi:10.1371/journal.pone.0318517)
Supplement: S1 Table — (DOCX) [file pone.0318517.s001.docx]

**Table S1.** Noise classes and the distance of temporary ponds to the two wind farms in Caetés, Pernambuco State, Brazil. For both wind farms, each class is represented by three temporary ponds. The table shows the range and average noise level per class, expressed in decibels, as well as the range and average distance between temporary ponds and wind farms, expressed in meters. Although not shown in the table below, the environmental noise in the control area was 67.7 dB.

| Sampled site | Noise class | Noise average (range) | Average distance (range) |
| --- | --- | --- | --- |
| Wind farm 1 | 1 | 122.9 (117.5-128.3) | 183.3 (52-274) |
| Wind farm 2 | 1 | 121 (114.6-127.4) | 116.6 (44-214) |
| Wind farm 1 | 2 | 112.1 (106.8-117.5) | 218.3 (109-388) |
| Wind farm 2 | 2 | 108.2 (101.8-114.6) | 141.6 (51-247) |
| Wind farm 1 | 3 | 101.4 (96-106.8) | 372.6 (73-873) |
| Wind farm 2 | 3 | 95.4 (89-101.8) | 460 (397-523) |
