# Supplementary material for: Wind farm noise negatively impacts the calling behavior of three frogs in Caatinga dry forests
Source: PLoS One. 2025 Mar 19;20(3):e0318517. doi: 10.1371/journal.pone.0318517 (PMC11922283; doi:10.1371/journal.pone.0318517)
Supplement: S2 Table — (DOCX) [file pone.0318517.s002.docx]

**Table S2.** Noise classes in temporary ponds distributed in two wind farms in Caetés, Pernambuco State, Brazil. For both wind farms, each class was represented by three temporary ponds. The table displays the range and average noise per class expressed in decibels (dB) with frequency weighting. Ranges, averages, and standard deviations are presented for spectral variables (Dominant Frequency, Frequency Range, and Call Amplitude), temporal variables (Call duration, Call Pulses, and Call Rate).

| Anuran species | Sampled site | Spectral variables | | | Temporal variables | | |
| --- | --- | --- | --- | --- | --- | --- | --- |
|  |  | Dominant frequency | Frequency range | Call amplitude | Call duration | Call pulses | Call rate |
| *Scinax pachycrus* | Wind farm 1 | 1895.1  (506.1-4026.8) | 3826.1  (2943-4540.2) | 82.2  (76.1-87.4) | 0.24  (0.18-0.28) | 22.5  (16.6-28.4) | 47.5  (33-65) |
|  | Wind farm 2 | 1702.7  (720-4049.4) | 3073.7  (2468.7-3543.3) | 78.6  (70.9-82.2) | 0.26  (0.17-0.39) | 22.1  (15.3-30.7) | 36.9  (24-49) |
|  | Wind farm 1 | 1799.4  (353-4151.2) | 3268.6  (2432.1-3943.9) | 80.5 (74.3-88.5) | 0.24 (0.14-0.32) | 20.35 (14.3-28.1) | 39.7 (21-51) |
|  | Wind farm 2 | 2614 (1704.5-4430.9) | 3587.8  (3029.6-4133) | 82.1  (75.1-88) | 0.25 (0.20-0.30) | 22 (17.9-28.6) | 45 (36-55) |
|  | Wind farm 1 | 1942.9  (362-3656.2) | 3595.7  (3014.7-4945.2) | 76.1  (72-81.5) | 0.26  (0.17-0.32) | 22.6 (15.5-30.9) | 43.4 (31-53) |
|  | Wind farm 2 | 1957 (122.3-3598.4) | 3314.5 (222-3361.9) | 71.8 (66.6-91.9) | 0.25 (0.19-0.32) | 21.83 (14.4-34.7) | 37.4 (21-49) |
|  | Less noisy area | 3409.1  (1619.3-4408.1) | 3833.8 (3253.7-4647.3) | 102.1 (88.2-110.2 | 0.27 (0.21-0.36) | 21.5 (17.5-24 | 40.9 (28-51) |
| *Scinax x-signatus* | Wind farm 1 | 775.7  (128.9-1861.1) | 2499.1 (1664.1-2986.1) | 76.3 (72-85.8) | 0.18 (0.13-0.22) | 9.74 (7.7-11.3) | 45.4 (22-62) |
|  | Wind farm 2 | 2076.5 (1477.9-2288.3) | 2890.6 (2625-3459.1) | 78.7 (68.5-87.8) | 0.17 (0.12-0.21) | 9.7 (7.2-11.6) | 42 (23-57) |
|  | Wind farm 1 | 1788 (51.5-3060.5) | 231.2 (1441.6-3412.5) | 81.9 (70.8-89.9) | 0.17 (0.14-0.20) | 9.6 (8.5-11.7) | 43 (23-56) |
|  | Wind farm 2 | 2435.7 (1201.4-3234.4) | 2864.7 (2527.8-3020.1) | 82.2 (77.4-87.4) | 0.18 (0.16-0.20) | 10.2 (9.2-11.8) | 47.7 (21-59) |
|  | Wind farm 1 | 698.7 (57.7-1568.2) | 2145.9 (1038.5-3107.2) | 76.9 (65.8-84.9) | 0.20 (0.16-0.24) | 10  (9-11.1) | 44.4 (33-56) |
|  | Wind farm 2 | 940.9 (178.6-2798.1) | 2631.1 (1858.7-3346.1) | 84  (76.1-87.1) | 0.18 (0.11-0.22) | 9.7  (7.8-11.9) | 34.4 (23-50) |
|  | Less noisy area | 1620.1 (358.7-5706.5) | 2959.4 (986.3-5738.4) | 85.9 (79.2-104.1) | 0.17 (0.12-0.22) | 9.9 (7.1-12.6) | 38.9 (23-52) |
| *Physalaemus*  *Cicada* | Wind farm 1 | 2500.2 (2447.5-2553) | 2421.7 (2351.4-292.1) | 76.6 (76.5-76.8) | 0.02 (0.02-0.02) | 6.9 (6.5-7.4) | 983 (883-1083) |
|  | Wind farm 2 | 2859.6 (2624.6-2961.5) | 1876.4 (1185.9-2744.6) | 79.6 (75.9-88.7) | 0.02 (0.01-0.02) | 5.9  (5.2-7.7) | 960.2 (565-1344) |
|  | Wind farm 1 | 2319.1 (664.2-2963.1) | 2329.6 (1755.1-2699.3) | 80.8  (69-86.2) | 0.02 (0.01-0.02) | 7.1  (5.8-8.1) | 992.1 (952-1260) |
|  | Wind farm 2 | 2863.8 (2773-2998.3) | 1641.3 (1129.7-2138.7) | 88.4 (86.2-91.9) | 0.02  (0.01-0.02) | 6  (4.8-6.9) | 1015.7 (703-1708) |
|  | Wind farm 1 | 2291 (346.3-2802.3) | 2085.9 (1636-2371) | 82.4 (77.5-91.7) | 0.01 (0.01-0.02) | 6.3 (5.2-7.6) | 873.8 (605-1173) |
|  | Wind farm 2 | 2764.7 (1965.6-3051.8) | 2253.1 (1694-2695.7) | 78  (73.1-82.9) | 0.02 (0.02-0.02) | 6.8 (5.6-7.7) | 803.6 (641-1032) |
|  | Less noisy area | 2764.7 (1965.6-3051.8 | 2253.1 (1694-2695.7) | 78  (73.1-82.9) | 0.02 (0.02-0.02) | 6.8  (5.6-7.7) | 803.6 (641-1032) |
