# Supplementary material for: Wind farm noise negatively impacts the calling behavior of three frogs in Caatinga dry forests
Source: PLoS One. 2025 Mar 19;20(3):e0318517. doi: 10.1371/journal.pone.0318517 (PMC11922283; doi:10.1371/journal.pone.0318517)
Supplement: S3 Table — (DOCX) [file pone.0318517.s003.docx]

**Table S3.** Pillai's test with significance of the relationship between wind turbine noise and advertisement call parameters of the species with their respective *F-values*, as well as the *P-value* of the MANCOVA.

| Anuran  Species | Treatment | MANCOVA | | | |
| --- | --- | --- | --- | --- | --- |
|  |  | Df | Pillai | F | P |
| *Scynax pachycrus* | Noise | 3 | 0.78 | 3.67 | >0.01 |
|  | Residual | 65 |  |  |  |
| *Scynax x-signatus* | Noise | 3 | 0.68 | 3.07 | >0.01 |
|  | Residual | 65 |  |  |  |
| *Physalaemus cicada* | Noise | 3 | 0.74 | 1.98 | >0.01 |
|  | Residual | 39 |  |  |  |
